# Longitudinal Evaluation of Cerebellar Signs of H-ABC Tubulinopathy in a Patient and in the *taiep* Model

**DOI:** 10.3389/fneur.2021.702039

**Published:** 2021-07-14

**Authors:** Milvia Alata, Arturo González-Vega, Valeria Piazza, Anke Kleinert-Altamirano, Carmen Cortes, Juan C. Ahumada-Juárez, Jose R. Eguibar, Alejandra López-Juárez, Victor H. Hernandez

**Affiliations:** ^1^Center of Research in Optics, Leon, Mexico; ^2^Department of Chemical, Electronic and Biomedical Engineering, Division of Sciences and Engineering, University of Guanajuato, Guanajuato, Mexico; ^3^Center of Rehabilitation and Child Inclusion Teleton, Chiapas, Mexico; ^4^Behavioral Neurophysiology Lab, Institute of Physiology, Benemérita Universidad Autónoma de Puebla, Puebla, Mexico; ^5^Research Office, Vicerrectory of Research and Postgraduate Studies, Benemérita Universidad Autónoma de Puebla, Puebla, Mexico

**Keywords:** H-ABC, tubulinopathy, quantitative MRI, segmentation (image processing), cerebellum, myelin, demyelination, ataxia

## Abstract

Hypomyelination with atrophy of the basal ganglia and cerebellum (H-ABC) is a central neurodegenerative disease due to mutations in the tubulin beta-4A (TUBB4A) gene, characterized by motor development delay, abnormal movements, ataxia, spasticity, dysarthria, and cognitive deficits. Diagnosis is made by integrating clinical data and radiological signs. Differences in MRIs have been reported in patients that carry the same mutation; however, a quantitative study has not been performed so far. Our study aimed to provide a longitudinal analysis of the changes in the cerebellum (Cb), corpus callosum (CC), ventricular system, and striatum in a patient suffering from H-ABC and in the *taiep* rat. We correlated the MRI signs of the patient with the results of immunofluorescence, gait analysis, segmentation of cerebellum, CC, and ventricular system, performed in the *taiep* rat. We found that cerebellar and callosal changes, suggesting a potential hypomyelination, worsened with age, in concomitance with the emergence of ataxic gait. We also observed a progressive lateral ventriculomegaly in both patient and *taiep*, possibly secondary to the atrophy of the white matter. These white matter changes are progressive and can be involved in the clinical deterioration. Hypomyelination with atrophy of the basal ganglia and cerebellum (H-ABC) gives rise to a spectrum of clinical signs whose pathophysiology still needs to be understood.

## Introduction

Hypomyelination with atrophy of the basal ganglia and cerebellum (H-ABC) is a neurodegenerative disease caused by mutations in the gene of TUBB4A (1–3). The effects of the mutations primarily affect the nervous system and cause several cerebral malformations (4). Tubulin has a very conserved sequence, and alpha-beta dimers assemble into microtubules that form the scaffolding for cell shape and intracellular movements (5). In humans, the tubulin superfamily includes more than 20 genes, 10 of which encode beta tubulins (6). Among the various isoforms, tubulin beta-4A (TUBB4A) represents 46% of all tubulins in the brain, with the highest expression in the cerebellum, putamen, and supratentorial white matter (7). In addition to the profound consequences on neural development and on the CNS white matter, the effects on mutations in the TUBB4A gene (8) show a range of clinical and radiological manifestations that depend on the identity and the position of the mutated residue and, possibly, on the cell type involved (9). The onset of the clinical findings is usually early in the infancy and includes motor development delay, pyramidal and extrapyramidal movements, ataxia, spasticity, dysarthria, and cognitive and sensory deficits (1, 10–13). Brain MRI shows myelin deficiency, involving the supratentorial white matter, CC, and internal capsule. The cerebellum and caudate-putamen are atrophic (1, 10, 12, 14–16). These changes can easily be associated with the characteristic dystonia, tremor, and ataxia described for most of the H-ABC patients. Similar findings have been reported in other diseases [for review, see (17)].

We have previously reported a case of a patient diagnosed with H-ABC carrying the Asp249Asn mutation in TUBB4A (13). Here, we analyzed by MRI segmentation the longitudinal changes of the affected structures in the central nervous system of the aforementioned patient and in the *taiep* rat, the only model of this disease that, as well as the patients, carries a spontaneous tubulin mutation (18). We used immunocytochemistry to correlate MRI findings to histological damage and analyzed physiological gait motor pattern and tremor signs. Finally, we also estimated the changes in other affected structures such as the CC and the ventricular system.

## Materials and Methods

### Case Presentation

We previously identified and confirmed a patient suffering from H-ABC (13). Briefly, a 3-year-old female was first admitted in the Department of Neurology due to motor signs. She presented a delay in the acquisition of developmental milestones, with global hypertonia. From the age of 6, she started with ataxia, dystonic postures in the extremities, action tremor, and progressive motor impairment. One year later, she lost all motor skills and dystonia was uncontrollable. She suffered two dystonic status events with oromandibular dystonia and lingual mutilation. Currently, severe dystonia is still present, which is treated with botulinum toxin twice a year.

### Genetic Data

As far as we know, inbreeding and isonymy are not present in the family history. We have previously shown that the patient carries the g.6366T>C and the g.6337G>A point mutations. While the first one is a silent mutation, the second substitutes an Asp with an Asn at position 249 at the amino acid level (13).

### Patient Magnetic Resonance Imaging

T1 (TR 500–567, TE 8.0–13.0)-, T2* (TR 3830–5270, TE 91.0–137.0)-, and T2-weighted fluid attenuated inversion recovery (FLAIR) (TI 2500, TR 8500, TE 105) magnetic resonance images were acquired at 5 and 11 years with a Siemens Magnetom Symphony (1.5 T). It was not possible to get digital MR images, so we digitized them from the radiological plates, taking care to acquire all images in the same conditions. All images were aligned to obtain a sequential stack of images.

### Patient and Rat Volumetric Brain Analysis

To manually segment 3D volumetric masks (cm^3^) for corpus callosum (CC), cerebellum (Cb), lateral ventricle (LV), and third and fourth ventricles, we used ITK-SNAP software (v.3.6.0; 1998–2017 US NIH) (17). We referred to a neuroanatomical atlas (19) as a reference of brain structures.

#### For the Patient

We manually segmented each structure of interest in each of the sagittal consecutive T2-weighted MRIs and extracted the whole brain. We designed a color code mask for each structure, i.e., pink for the cerebellum, cyan for CC, red for LVs, green for the third ventricle, and blue for the fourth ventricle. After that, we obtained measurements of each structure of interest using the ITK-SNAP volume estimation tool. The volume is calculated as a function of the number of voxels in each mask and then the volume of each voxel for both ages is analyzed. To have a proper understanding of the volume changes in the structures at the two ages, we normalized the analyzed structures to the cranial volume (%). Based on a previously reported method, we calculated the cranial capacity of the patient at 5 and 11 years old using three measurements: maximum head length (glabella-inion length), maximum head breadth (measured between parietal eminences), and the height between the highest point of vertex and the external acoustic meatus (20).

The changes in the different structures in function of age were calculated as the percent ratio of volume changes, between 11 and 5 years:

$$\begin{array}{l} \left(\left(V_{11\text{years}}-V_{5\text{years}}\right)/V_{5\text{years}}\right)\times 100. \end{array}$$

For comparison, we took the data for volumes per brain regions in normal children at 7 and 13 years, previously published (21), and made the same analysis we did for our measurements.

#### For *taiep* Rats

All animals were provided by the animal facility of the Benemérita Universidad Autónoma de Puebla where *taiep* rats were originally described and raised.

Volume analysis of the whole encephalon, the CM, Cb, LV, 3rd, and 4th ventricles was carried out using ITK-SNAP (V 3.6.0) software. The areas used to calculate volumes were obtained for each slice by manually tracing the anatomical structure's contour.

### Gait Analysis

The gait was recorded using the CatWalk 9.1 system (Noldus Technologies, The Netherlands); we recorded the number of complete stepping cycles, the recording time, and the regularity index that is the coordination between the limbs and their cadence.

The CatWalk system is a catwalk made of black Plexiglas. The roof consists of a red diode system and the platform is a 6-mm crystal illuminated with green light. The catwalk is 21 cm in length. The stepping was obtained through a fast speed camera (Gevicam model GP-2360C) and sent through a cable to a computer server Dell precision T3500.

For the analysis of the stepping and coordination among the four limbs, we used the CatWalk™ software v. 9.1 under Windows 7 software.

To obtain a regular stepping pattern, all subjects are trained three times daily for 3 days. We added to the CatWalk system an acrylic dark box (8 × 24.5 × 15 cm) to promote stepping to the end of the walking tract and we used Fruit Loops™ (Kellogg's company, México) as a reward.

The data were analyzed using GraphPad Prism (version 9.1.0 GraphPad Software Inc., La Jolla, CA, USA).

### Rat Magnetic Resonance Imaging: T2-Weighted Images

The *in vivo* acquisition study included six *taiep* and four Sprague–Dawley wild-type (WT) rats as controls at 1, 2, and 8 months. High-resolution images of male rats' brains were acquired using a Helium-cooled 7.0 T scanner (BRUKER PHARMASCAN 70/16 Billerica, MA, USA) equipped with a gradient set with Gmax = 760 mT/m. During the *in vivo* MRI recording, the animals were anesthetized with isoflurane (Sofloran, PiSA Mexico), 5% concentration dose for induction, and 1–2% to maintain an adequate anesthesia level. A pulse oximeter monitored the rats' oxygenation level, and the body temperature was kept constant throughout the experiment using a thermoregulated water circulation system. T2-weighted images were acquired with contiguous 0.8-mm sections in the coronal plane using a Rapid Acquisition with Refocused Echoes (RARE) sequence with the following parameters: repetition time (TR) of 2,673.5 ms; echo time (TE) of 33 ms, field of view (FOV) 20 × 18 mm^2^, matrix size 200 × 180 corresponding to an in-plane resolution of 0.1 × 0.1 mm^2^. T2-weighted images of sagittal and axial sections were acquired using a sequence with a TR of 15 ms, TE 3 ms, FOV 100 mm^2^, and an in-plane resolution of 0.208 × 0.208 mm^2^.

### Tissue Preparation and Immunocytochemistry

Rats were anesthetized with a mixture of ketamine–xylazine (0.125–5 mg/kg, IP) and then sacrificed by decapitation. Brains and cerebella were immediately fixed in 4% formaldehyde in PBS for immunohistochemical processing. Fixed tissue sections were immersed in 30% sucrose in PBS at 4°C for 24 h and frozen using tissue freezing medium (ref. 14020108926, Leica, USA). Thirty-micron slices were obtained in a CM 1860 cryostat (Leica, USA).

Sections were marked with an anti-neurofilaments 200 antibody (N4142, Sigma, USA) and immunostained with Alexa Fluor 488 (A11070, Thermo Fisher Scientific, USA). Myelin sheaths were stained with Fluoromyelin red (F34652, Thermo Fisher Scientific, USA) as indicated in the datasheet. Nuclei were stained with DAPI (62248, Thermo Fisher Scientific, USA).

### Microscopy

Immunofluorescence images were acquired with an LSM-710 confocal microscope (Zeiss) equipped with an LCI Plan-Neofluar 25X/0.8 and an alpha Plan-Apochromat 63×/1.46 Oil Korr M27 immersion objectives.

Pseudo-bright-field images were generated with residual 405-nm laser light captured in transmission by an external non-descanned detector (NDD) of the LSM 710 Zeiss confocal microscope, using a 25× oil-immersion objective. Bright-field images of whole organs were acquired with a Cytation 5 cell imaging multi-mode reader (Biotek, Vermont, U.S.A.) with a 4× objective. FIJI software (Schindelin, J, 2012) was used to convert and reconstruct the fluorescence and bright-field images.

### Statistics

Statistical analyses were carried out with GraphPad (version 9.1.0 GraphPad Software Inc., La Jolla, CA, USA). Data are plotted as mean ± SEM. Points represent individual measurements in at least *n* = 3 per group. Statistical differences were analyzed with appropriate tests, indicated in figure legends, comparing the WT and *taiep* groups. For all experiments, *p* < 0.05 was considered significant.

### Institutional Review Board Statement

All experimental procedures were carried out following the rules of the Declaration of Helsinki of 1975 revised in 2013 and in compliance with the laws and codes approved in the seventh title of the regulations of the general law of health, regarding health research, of the Mexican government (NOM-033-Z00-1995 and NOM-062-ZOO-199) and in accordance with the recommendations of the National Institutes of Health Guide for the Care and Use of Experimental Animals (eighth edition, 2011). All the procedures for animals and patients were approved by the institutional committee of bioethics in research of the University of Guanajuato and the Benemérita Universidad Autónoma de Puebla.

Even though this work does not include any participation or interaction with the patient, permission to use the MRIs was granted from the patient family.

## Results

### Cerebellar and Callosal Atrophy in the Patient Suffering From H-ABC

**Figure 1:**
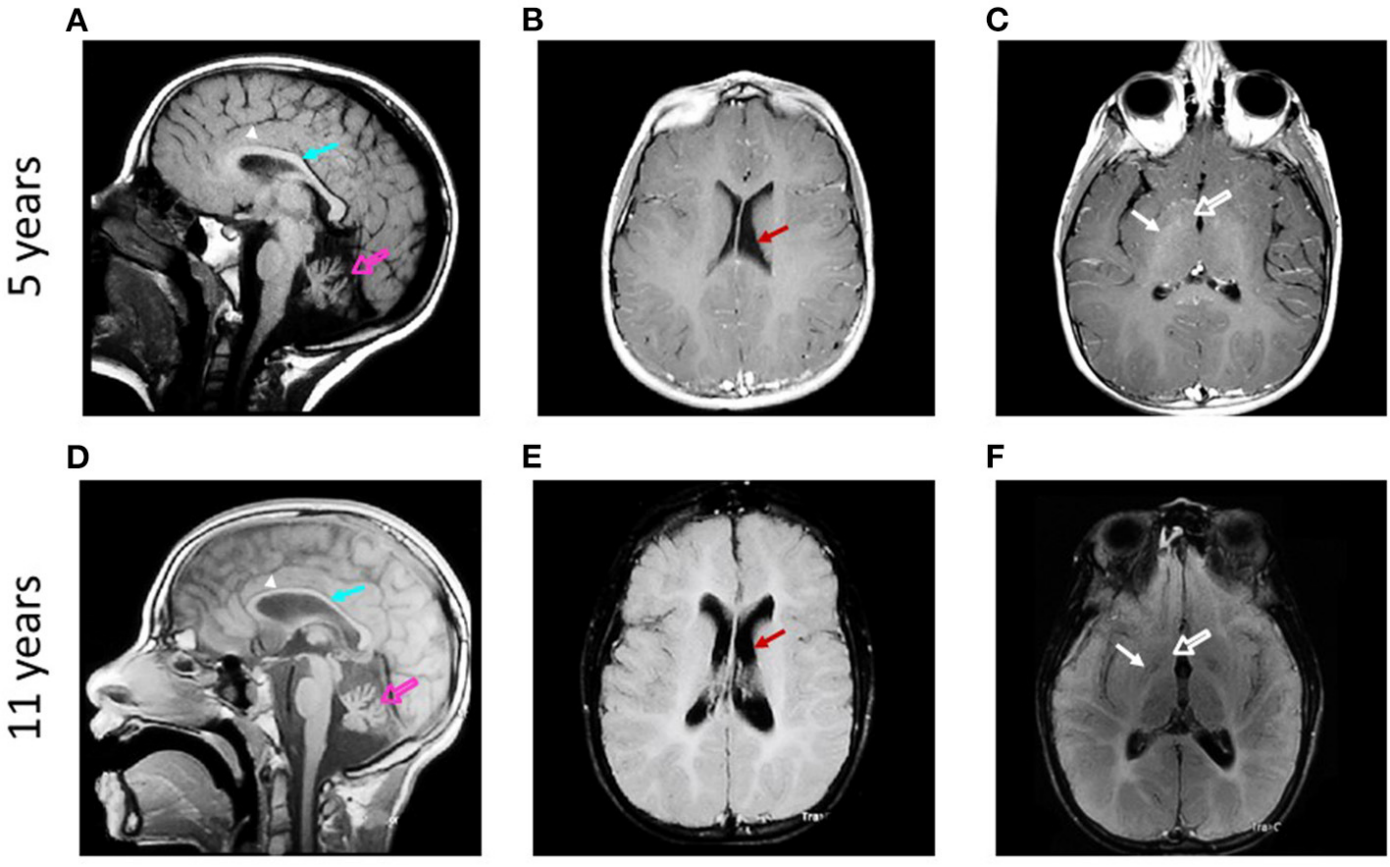
Qualitative analysis of the evolution of brain structures in a patient suffering from H-ABC. Sagittal T1-weighted magnetic resonance images obtained at 5 **(A)** and 11 years **(D)** show atrophy of cerebellum (open arrows) and corpus callosum (arrows) and involvement of U-fibers (arrowheads). Axial T2-FLAIR-weighted magnetic resonance images obtained at 5 **(B,C)** and 11 years old **(E,F)** show atrophy of the brain cortex and ventriculomegaly [arrows in **(B,E)]**. Atrophy of caudate (open arrows) and putamen (arrows) is also observed **(C,F)**.

We analyzed the brain MRIs of the patient at two developmental ages, 5 and 11 years. The first magnetic resonance scanning was done at the age of 5, in the three planes (Figures 1A–C). Six years later, a second comparative MRI was performed (Figures 1D–F). Hypointensity in sagittal T1-weighted images and hyperintensity of the white matter in the ventricles' contours in axial T2-fluid-attenuated inversion recovery (FLAIR) suggested a potential hypomyelination at both ages (Figures 1A,F). Magnetic resonance angiography was normal (data not shown). T1 images showed cerebellar atrophy and hyperintensity of the deep white matter of the brain, with involvement of U-fibers (Figures 1A,D). T2-FLAIR images confirmed these findings. Furthermore, comparing the hyperintensities in the cerebellum and CC at both ages, it was possible to observe a progressive loss of white matter. Pathognomic radiological signs of atrophy of the brain cortex, CC, cerebellum, and basal ganglia were evident at both ages (Figure 1). Sagittal and axial projections showed hydrocephalus *ex vacuo* and asymmetric ventriculomegaly, worsening with the age of the patient (Figure 1 and Supplementary Videos 1–4).

**Figure 2:**
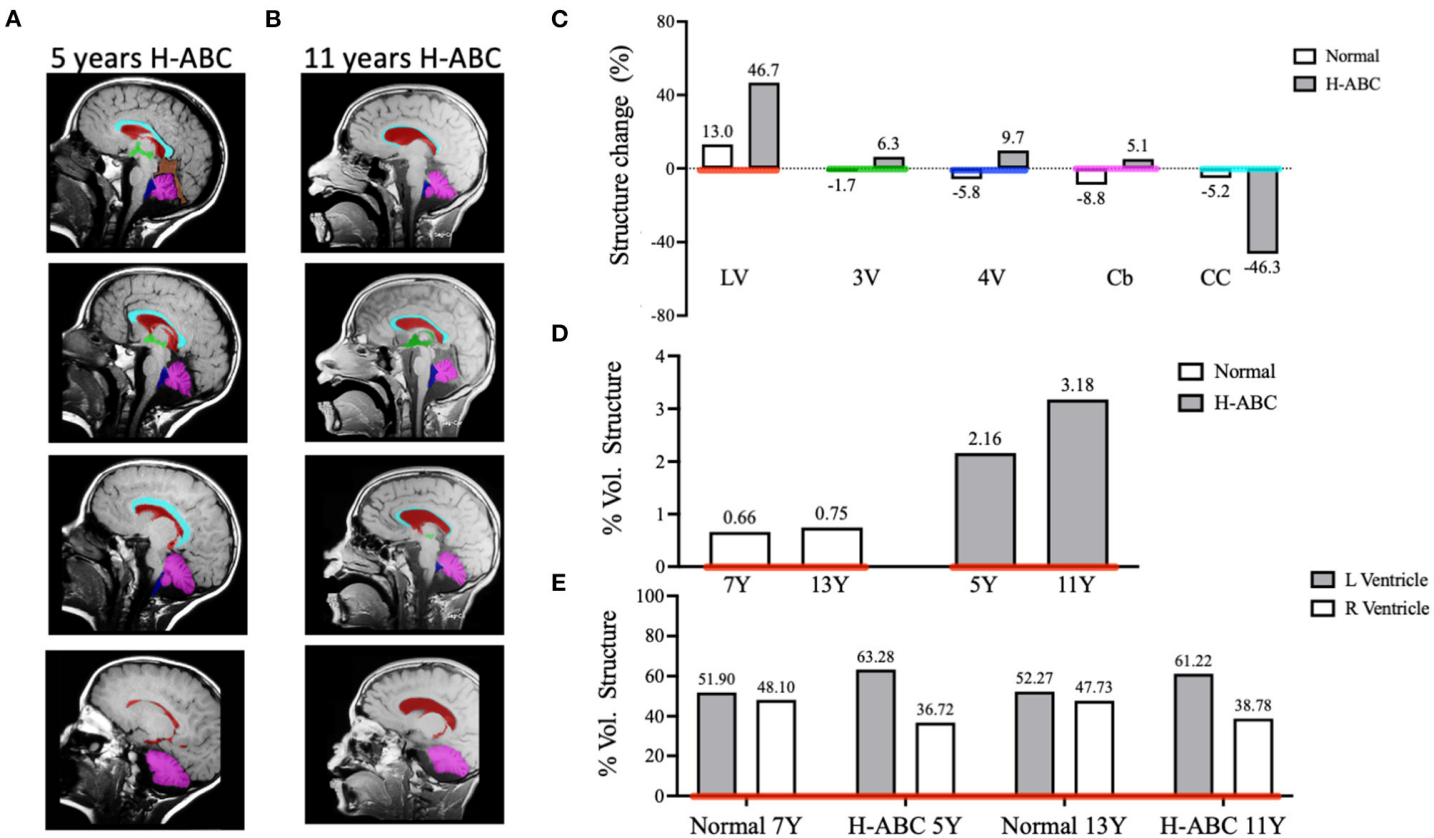
Comparative analysis of brain structure volumes in the H-ABC patient and normal children in a period of 6 years. Representative images of T1-weighted sagittal sections illustrating individual masks in the brain structures of interest **(A,B)**. Lateral ventricles (LV) in red, third ventricle (3V) in green, fourth ventricle (4V) in blue, cerebellum (Cb) in magenta, and corpus callosum (CC) in cyan. Change of volume structures from normal children and the H-ABC patient **(C)**. The percentage of volume of the lateral ventricles related to the cranial volume **(D)**. The percentage of left and right lateral ventricles related to total lateral ventricle volume **(E)**. Numbers at the top of each bar indicate the value of represented data.

To evaluate volume changes, we performed a semi-quantitative analysis of cerebral structures using image segmentation analysis. The manual segmentation of each structure of interest was highlighted with a different color mask and then isolated to calculate the cranial volumes at both ages (Figures 2A,B). The patient's cranial volume was estimated using anatomical measurements of the cranial structures obtained from the axial and sagittal MRIs. The measures obtained at 5 years were maximum head length 16.91 cm, vortex-acoustic meatus 12.47 cm, and maximum head breadth 13.67 cm, and those obtained at 11 years were maximum head length 17.53 cm, vortex-acoustic meatus 12.32 cm, and maximum head breadth 13.79 cm. The calculated cranial volumes were 1,110.43 and 1,142.33 cm^3^ for 5 and 11 years, respectively. Next, we quantified the volume of the cerebellum and CC, as well as those of the third, fourth, and LVs.

The volume of each structure was expressed as a percentage of structure volume change calculated as described in *Materials and methods*. When comparing the volume change in a period of 6 years of H-ABC vs. normal children (21), we observed an enlargement of the LVs (46.7 vs. 13.0%), 3rd ventricle (6.3 vs. −1.7%), 4th ventricle (9.7 vs. −5.8%), and cerebellum (5.2 vs. −8.8%); on the other hand, the CC decreased its volume (−46.3 vs. −5.2%) (Figure 2C). Moreover, the ventriculomegaly (2.164 vs. 3.175%) and CC atrophy (0.703 vs. 0.377%) observed in the qualitative analysis were confirmed also with quantitative data (Figure 2C).

The progressive enlargement of the LV in H-ABC expressed as volume related to the cranial volume was 2.6–3.18%, while in normal children, it goes from 0.66 to 0.75% in the same period (Figure 2D).

Qualitatively, it was evident that the left ventricle was larger than the right one. Indeed, when quantified, left ventricle was always larger than the right ventricle at both analyzed ages (63.28 vs. 36.72 and 61.22 vs. 38.78%, respectively). While in normal children, left and right ventricles are symmetric (51.9 vs. 48.1 and 52.27 vs. 47.73%, respectively) in the same period (Figure 2E).

### Ataxic Gait Analysis in the *taiep* Rat

**Figure 3:**
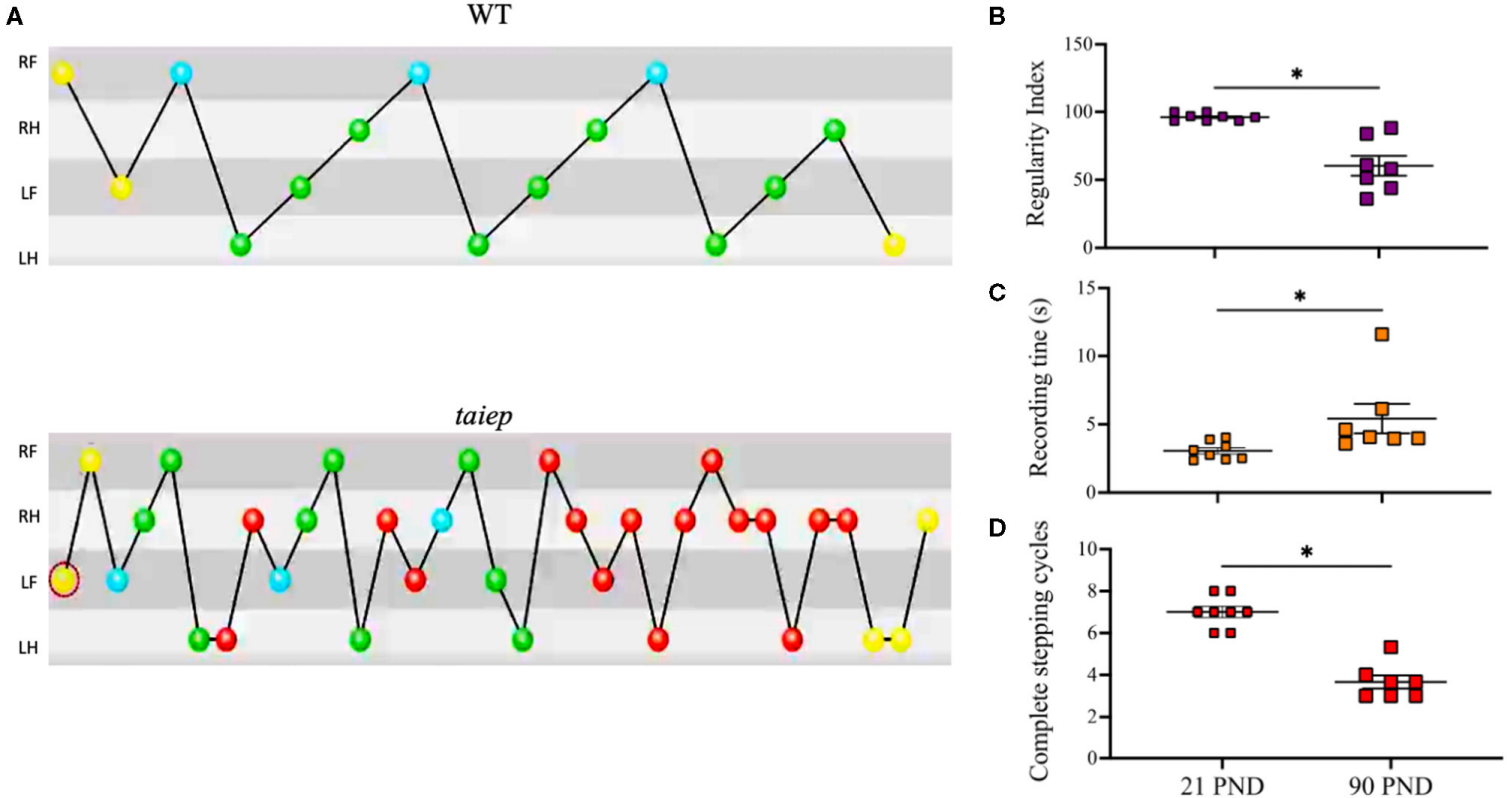
Analysis of ataxic gait in the *taiep* rat. Stepping pattern and limb coordination shows that *taiep* rats performed more steps of short distance to diminish ataxic gait to be able to cross the catwalk **(A)**. The regularity index decreases with the age of *taiep* rats **(B)**. The recording time increases **(C)** and the number of complete stepping cycles decreases **(D)** with the age of *taiep* rats (PND: postnatal days). Yellow circles: not taken into account; blue circles: start of a pattern; green: part of a pattern; red: not part of a pattern. Data were analyzed by Wilcoxon matched-pairs signed rank test. *p > 0.05 between groups. RF, Right Front; RH, Right Hind; LF, Left Front; LH, Left Hind.

Gait coordination analysis was made using the CatWalk system (Noldus Technologies, The Netherlands) (Figure 3A and Supplementary Videos 5, 6). The results showed a significant decrease in the number of complete stepping cycles from 21 to 90 postnatal days (*p* < 0.05) (Figure 3B). The duration of the stepping pattern along the catwalk significantly increases with the age of the subjects (*p* < 0.05) (Figure 3C). Importantly, the regularity index, which is a measure of the coordination among the four limbs, significantly decreased with age (*p* < 0.05) (Figure 3D).

### Longitudinal Quantitative Analysis of Magnetic Resonance Images in the *taiep* Rat

**Figure 4:**
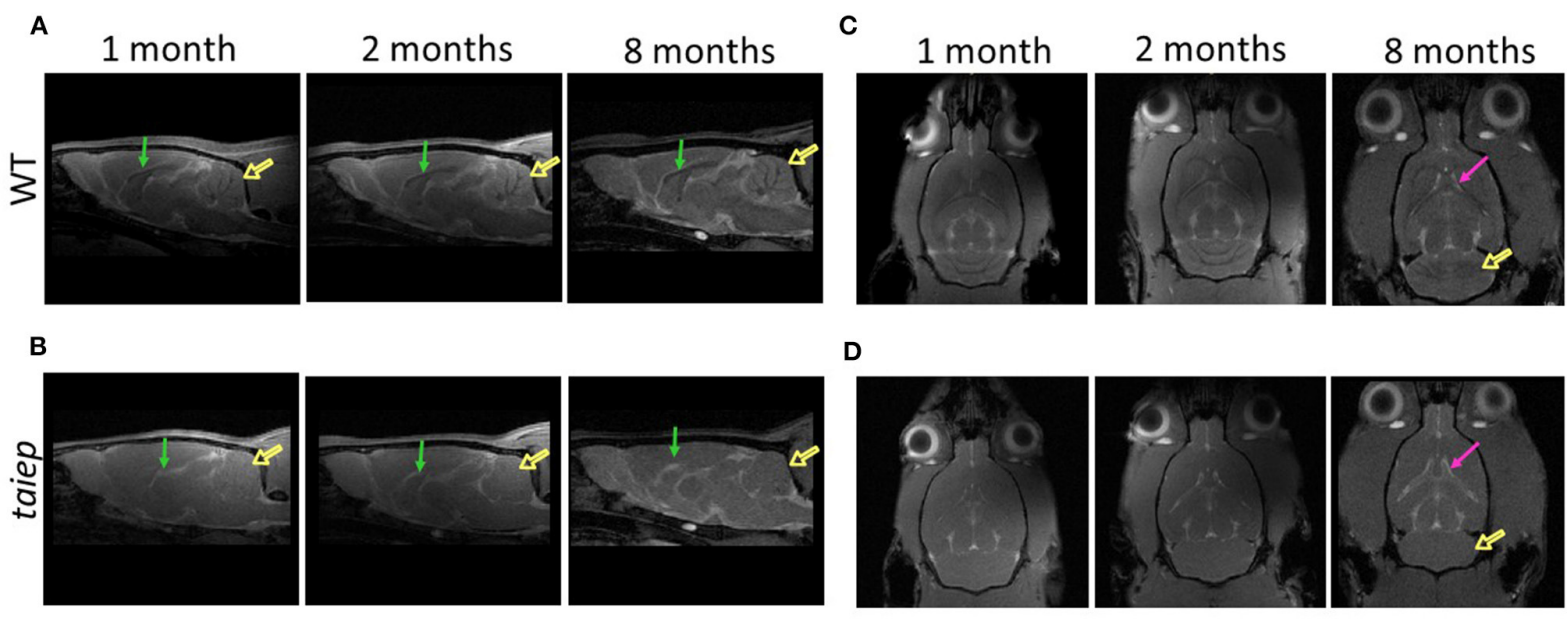
Comparative analysis of the development of brain structures in the *taiep* rat. Representative images of sagittal T2-weighted MRIs obtained at 1, 2, and 8 months for the wild-type and *taiep* rat **(A,B)**. Signals of the white matter of cerebellum (open arrows) and corpus callosum (arrows) are less visible in *taiep* rats compared to WT. Representative axial T2-weighted MRIs obtained at 1, 2, and 8 months **(C,D)**. Progressive enlargement of lateral ventricles (arrows) and cerebellar demyelination (open arrow) is observed in *taiep* rat at 8 months **(C,D)**.

Sagittal and axial T2-weighted images of the same *taiep* rats were acquired at 1, 2, and 8 months. WT and *taiep* rats were scanned in the same session and at the same ages (Figure 4). The high resolution of the images made it possible to measure the volume of the cerebellum (Cb), CC, LV, CC, LV, and the whole encephalon. White matter tracts in control WT rats were hyperintense (Figures 4A,C). On the other hand, in the *taiep* rat, the cerebellar and callosal white matter generated hypointensities, consistent with the loss of lipid content (Figures 4B,D). When comparing *taiep* MRIs at different ages, it is possible to observe that the ventricular volume increases with age.

### Analysis of Cerebellar Volume Shows Severe Cerebellar Demyelination Without Changes in the Total Volume

**Figure 5:**
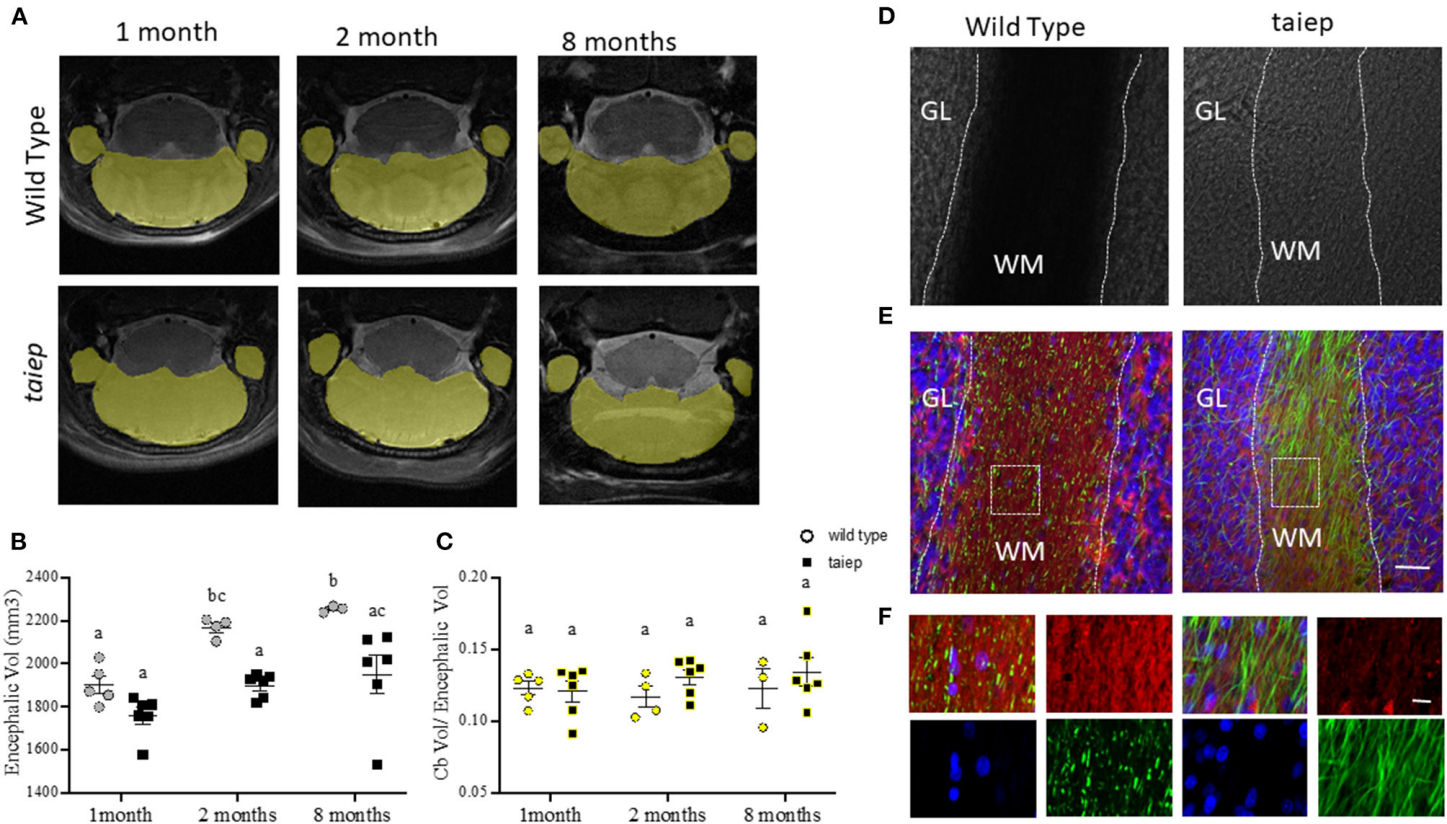
Anatomical and histological analysis of cerebellum in *taiep* rat at different stages of development. Representative images of T2-weighted coronal sections illustrating individual masks in cerebellum (yellow) at 1, 2, and 8 months **(A)**. Scatter plot of the values of encephalic volume. WT encephalon volume was significantly bigger than that of *taiep* rats at 2 and 8 months **(B)**. Non-significant differences were obtained for volume ratio cerebellum/encephalon of WT and *taiep* rats along the analyzed periods **(C)**. The data obtained for each rat are represented by one point in the plot (at least *n* = 3 for group). Two-way ANOVA followed by Tukey's multiple comparison test was performed. Different letters indicate significant differences between groups **(B,C)**. Bright-field micrographs of coronal slices of cerebellum of WT and *taiep* rat brains (10 months) **(D)**. White dotted lines delimitate the white matter region in cerebellar folia. Myelin (red) and neurofilament (green) were revealed by immunofluorescence **(E,F)**. An enlarged view of the white squares for merge and separated channels is shown in **(F)**. Nuclei were revealed by DAPI (blue). Scale bars: 30 μm **(E)** and 10 μm **(F)**. GL, granular layer; WM, white matter.

The high resolution of our T2-weighted images made it possible to perform precise segmentation of the cerebellum (Figure 5A). To calculate the longitudinal cerebellar volume changes, they were related to the whole brain volume changes. As expected, in WT rats, the encephalic volume increased significantly (*p* = 0.0283) from 1 month (1,900.2 ± 40.4 mm^3^) to 2 months (2,168.1± 22.3 mm^3^) and then remain unchanged (*p* = 0.933) until 8 months (2,253.3 ± 8.5 mm^3^). Instead, in the *taiep* rat, the encephalic mass did not show the same growth pattern, i.e., no significant (*p* = 0.348) increase of volume was observed at any analyzed age (to 1 month: 1,757.5 ± 38.6 mm^3^; to 2 months: 1,898.1 ± 22.7 mm^3^; and to 8 months: 1,949.8 ± 89.8 mm^3^). Comparing WT and *taiep* rats, the encephalic volume did not show significant differences at 1 month of age (*p* = 0.383). However, a delay in the growth rate in *taiep* rats is statistically significant at 2 and 8 months compared to WT volumes (*p* = 0.0197 and 0.0159, respectively) (Figure 5B).

WT and *taiep* rat's cerebellar volumes were calculated in the same animal samples where we had previously measured the encephalic volumes. Cerebellar volumes were 233.5 ± 11.5 vs. 212.48 ± 14.5 mm^3^ at 1 month; 253.4± 16.7 vs. 248.25 ± 11 mm^3^ vs. at 2 months, and 275.4 ± 30.1 vs. 258.6 ± 14.3 mm^3^ at 8 months, for WT and *taiep*, respectively. Considering the delay in encephalic growth in *taiep* rats, we calculated the ratio between the cerebellar and encephalic volumes. We did not find significant differences (*p* > 0.05) at the three ages between those ratios, either for control or *taiep* rats (Figure 5C).

We used the same brains of rats analyzed by MRI to perform the histological analysis of the structures of interest, including the cerebellum in *taiep* and WT rats of the same age (10 months). In bright-field micrographs, the white matter of WT rats has a higher optical density than the granular layer (Figure 5D). On the other hand, the white matter of *taiep* rats looks less dense. The fluorescent myelin staining is abundant and compact in WT rats while it is sparse in *taiep* rats (Figures 5E,F). Furthermore, neurofilament immunofluorescence reveals the demyelinated axons (Figures 5E,F). These histology findings are consistent with the hyperintensities observed in cerebellar MRIs of *taiep* rats and confirm the damage in the myelin of the cerebellar white matter.

### Ventriculomegaly Is Also Observed in *taiep* Rats

**Figure 6:**
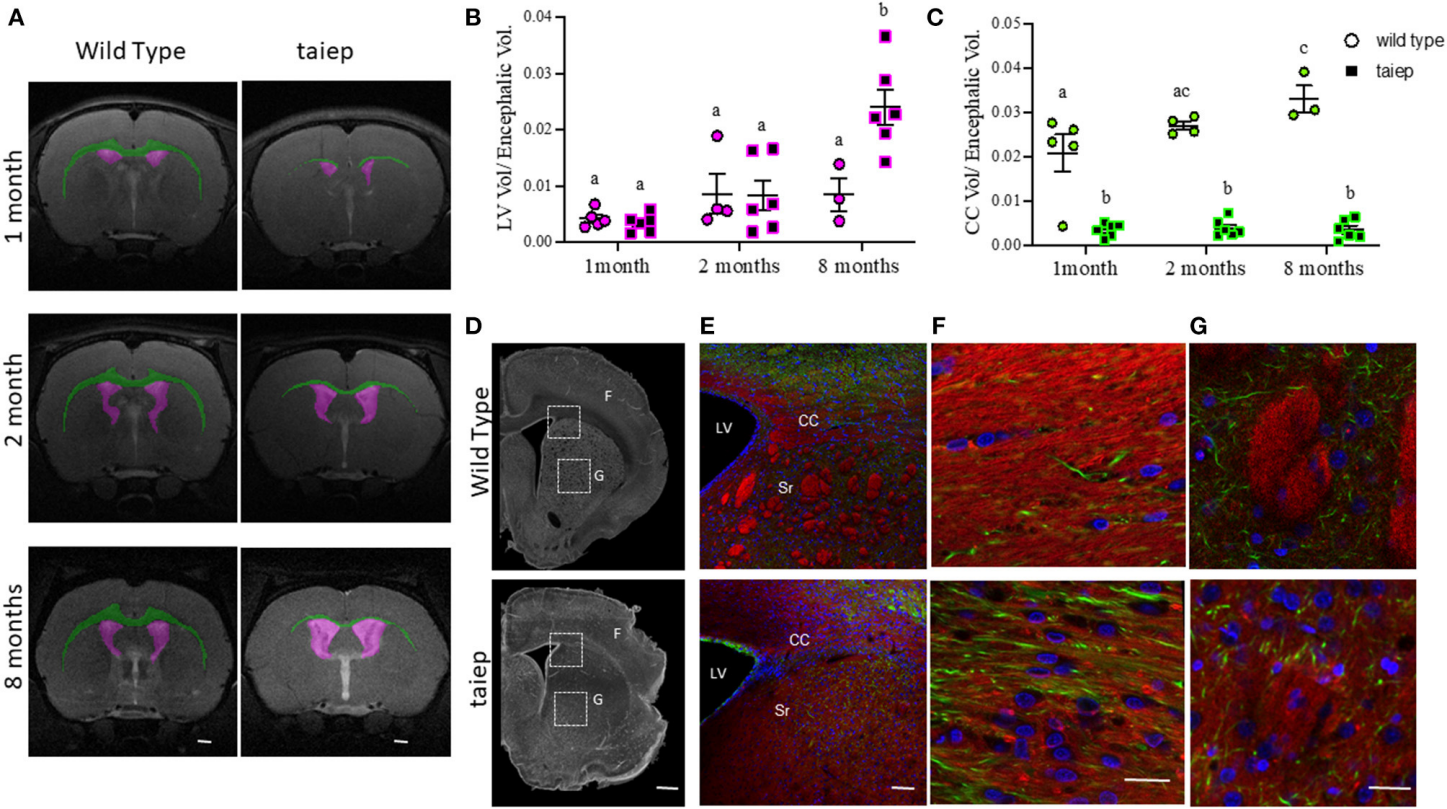
Anatomical and histological matched analysis of corpus callosum in *taiep* and wild-type rats at different stages of development. Representative images of T2-weighted coronal sections illustrating individual masks in the brain structures of interest at 1, 2, and 8 months. Lateral ventricles in magenta and corpus callosum in green **(A)**. The volume ratio was calculated as structure volume/encephalon volume (in mm^3^) at the same age **(B,C)**. Scatter plot of the values of LV volume ratio **(B)** and CC volume ratio **(C)**. Each dot represents the data from one rat (at least *n* = 3 for each group). Two-way ANOVA followed by Tukey's multiple comparison test was done. Different letters indicate significant differences between groups. Representative coronal slices of corpus callosum and striatum of WT and *taiep* rat brains (10 months) acquired by phase contrast microscopy **(D)**. Myelin (red) and neurofilaments (green) were revealed by immunofluorescence **(E–G)**. Amplifications of white squares for merge and separated channels are shown in **(F)**. Striatum **(G)**. Nuclei were revealed by DAPI (blue). Scale bars: 1,000 μm **(D)**, 20 μm **(E)**, 5 μm **(F)**, and 100 μm **(G)**. LV, lateral ventricle; CC, corpus callosum; Sr, striatum.

As in the patient, MRI coronal sections of rat brains suggest changes in the CC and LV of *taiep* rats during development (Figure 6A). We analyzed the volume of these structures in a longitudinal study at 1, 2, and 8 months.

The volume of LVs did not show significant differences (*p* > 0.05) at the analyzed ages (8 ± 1.4 mm^3^ and 18.7 ± 7.6 mm^3^) in WT rats. Instead, in the *taiep* rat, even though there were no significant changes in the volumes of the LVs (6 ± 1.2 and 15.5 ± 4.8 mm^3^) between the first and second month (*p* = 0.801), a dramatic increase in their volume appeared at 8 months (45.7 ± 4.4 mm^3^) (*p* < 0.0001 comparing 1 vs. 8 months and *p* < 0.0002 comparing 2 vs. 8 months) (Supplementary Figure 2). Related to the encephalic volume, ventriculomegaly was significant in the *taiep* rat (*p* < 0.0001 comparing 1 vs. 8 months; *p* = 0.0007 comparing 2 vs. 8 months) at 8 months (Figure 6B).

### Analysis of Corpus Callosum Atrophy in *taiep* Rats by MRI and Histology

The CC of WT rats was easily recognizable in the brain's MRIs (Figure 6A, green), while in the *taiep* rat, the CC appeared very thin in some sections or even indistinguishable in others (Figure 6A, green). We measured the volumes of the CC in the regions where it was visible in the T2-weighted images. In WT rats, the CC volume increased significantly from 1 to 2 months (39.4 ± 7.95 and 58.4 ± 1.8 mm^3^, respectively; *p* = 0.03) and from 1 to 8 months (74.3 ± 7 mm^3^, *p* = 0.0001). However, we did not find significant differences (*p* > 0.05) when comparing CC volume from 2 to 8 months. There were no significant differences in *taiep* rats when the volume of the CC was compared at different ages (at 1 month: 6.06 ± 1.1 mm^3^; at 2 months: 7.3 ± 1.5 mm^3^; and at 8 months: 6.6 ± 1.7 mm^3^) (Supplementary Figure 2).

Considering the differences in the encephalic volume between WT and *taiep* rats, we calculated the CC volume related to the encephalic volume. Data obtained showed that the CC volume of WT rats increased proportionally to the encephalic volume. We found significant differences between 1 and 8 months (*p* = 0.010), but not between 1 and 2 months or 2 and 8 months, suggesting that CC grows slowly during WT rat development. Instead, in the *taiep* rat, the volume of the CC related to the encephalic volume did not show significant differences (*p* > 0.05) at any of the analyzed ages, indicating that the CC is atrophic and it does not grow during development (Figure 6C). It is important to note that the CC volume related to the encephalic volume in *taiep* rats is significantly smaller (*p* < 0.0001 for compared ages) than that observed in WT rats at all the analyzed ages.

A possible explanation for these findings is that the CC is atrophic due to demyelination. To corroborate this hypothesis, we analyzed the CC in fixed tissue sections (Figures 6E–G). In phase-contrast micrographs, the white matter of CC of WT rats was optically denser than in *taiep* rats (Figure 6D). A dimmer fluorescent myelin staining in *taiep* rats confirmed the damage in the white matter (Figures 6E,F). Additionally, we observed that the same demyelination affected striosomes of *taiep* rats (Figure 6G).

## Discussion

In this work, we analyzed longitudinal changes in cerebellar volume and white matter characteristic structure, both in a patient and in the rat model of H-ABC, combining clinical data, physiological analysis of the rat's ataxic gait, segmentation of MRIs, and immunohistochemistry. We also analyzed the changes in some other structures involved in this tubulinopathy.

Genetic analysis is used to confirm the clinical and radiological findings in patients suffering from H-ABC; nevertheless, to this day, the causative relationship between the symptoms and the underlying mutations is still very complicated due to the relatively recent description of the disease and the incomplete understanding of each tubulin mutation's effects on the microtubules and cell physiology. Furthermore, due to the unavailability of pathology material and to ethical reasons, it is challenging to study pathophysiology of H-ABC in humans. For these reasons, an animal model is invaluable to investigate the disease's mechanisms and essential for the proposal of therapeutics. The *taiep* is a tubulin mutant that shares clinical and radiological signs with human patients (18).

Our patient carries the point mutation D249N (13). Even though this mutation is commonly found in this kind of tubulinopathies (22–25), the clinical and radiological FLAIR signs could vary among patients (9, 11, 24, 26). Our work's relevance stems from the quantitative longitudinal analysis of the neurodegenerative changes in our patient's brain, which were also correlated to the same changes in *taiep* rats.

In the case of the patient, imaging analysis is based on the changes in brain structures related to skull volume. Recently, Mongerson et al. reported changes in the CC employing a method similar to the one we used here to evaluate degenerative processes in the brain (27). They normalized the quantitative differences in the CC (in %) using total brain tissue. In our case, it was not possible to use total brain volume because of changes in whole brain mass due to the neurodegenerative process (11). These changes are quite evident in our patient, e.g., we observed a progressive increase in the ventricular system volume and hydrocephalus *ex vacuo*. Consequently, the brain volume cannot be used as a fixed parameter to compare volume changes in deep brain structures. Instead, we used a previously described method (20) to normalize the volume of the analyzed structures to the cranial capacity.

Several works describe qualitative comparisons of the changes between MRIs at different ages in the same patient. For example, Hamilton et al. analyzed 42 patients with mutations in TUBB4A (22). They found varying levels of hypomyelination and atrophy of basal ganglia and cerebellum, with only one patient not developing cerebellar atrophy. In some of their patients, there was severe dilatation of the third and LVs. Joyal et al. compared MRIs at 6, 11, and 17 months in the same patient(s), finding no progression of myelination, and progressive atrophy of the caudate, putamen, cerebellum, cerebral hemispheres, and CC (22). We did not detect progressive atrophy of the cerebellum, either in the patient or in *taiep* rats. It is important to note that at a first glance, the cerebellum of our patient is larger than the cerebellum in normal children. However, a deeper analysis considering the cerebellar volume related to the cranial volume clearly shows that the cerebellum in our patient is approximately 50% smaller than in normal patients (5.25% at 11 years vs. 10.46% at 7 years, respectively), and the change in cerebellar volume is minimal (0.26 vs. 0.92%).

Early anatomical descriptions of the *taiep* rat showed that the cerebellum seems to be atrophic as its weight is 16% lower than in the WT rat (28) and qualitative analyses showed that the cerebellum was hypomyelinated (29) but without a neuronal loss (30). Indeed, our immunofluorescence data confirmed a normal cellular distribution but severe damage of the white matter as a consequence of hypomyelination and a progressive demyelination. This progressive white matter loss, without apparent involvement of other cerebellar regions, could well explain the decrease in the stepping pattern and the ataxic gait.

Cerebellar ataxia can be a clinical manifestation of several genetic diseases (31–33), among them some leukodystrophies (34, 35). So far, it is not known how tubulin mutations induce cerebellar atrophy; however, in this organ, the ataxic signs of H-ABC due to myelin loss could be explained with the damage on afferent and efferent pathways. Nevertheless, motor signs of this disease cannot be explained only with cerebellar dysfunction. Contrary to what was previously suggested (30), we found clear evidence of striatal damage in the *taiep* rat. MRI shows a poor definition of this structure even in very young animals (18). Here, we confirmed this damage by phase-contrast and confocal microscopy. Motor dysfunction reflects damage in a nervous network that involves also basal ganglia and cortical areas (36–38).

Clear evidence of the progression of the demyelination process is the atrophy of the CC. Our volumetric analysis revealed that this structure lost 46% of its volume between ages 5 and 11. As in other reported cases, our patient started with a delay in motor development, which evolved into ataxia, dystonia, tremor, and progressive motor deterioration. With the rest of the degenerative process, the dramatic change in the CC can contribute to the motor consequences of this pathology and account for the patients' cognitive deficit.

Finally, LVs drastically increased their volume by 46.7% between 5 and 11 years in the patient. By contrast, the third and fourth ventricles' volume ratios were minimal during this developmental period (6.3 and 9.7%, respectively). The progressive enlargement of LVs has been reported before (10–12, 22), but it has never been quantified. Most probably, it is due to the atrophy of white matter over time (10). There are no conclusive explanations in the literature for this swelling, but other authors have also found the same observation in H-ABC tubulinopathy (11, 12). It must be emphasized that, in our patient, the increase in the LV volume is asymmetric; i.e., the left ventricle is larger than the right one (63.28 and 36.7%, respectively). Asymmetric ventriculomegaly is also present in other brain malformations, and it has been associated with white matter injury (39–41).

Our longitudinal volumetric analysis shows the changes in different central structures in a patient suffering from H-ABC and in the animal model of this disease. Our results showed that cerebellum atrophy does not progress during development. A possible hypothesis is that this atrophy develops during the intrauterine life due to still unknown mechanisms, but it does not advance after birth. Still, white matter damage progresses, and hydrocephalus *ex vacuo* is a consequence of this neurodegeneration. All this could explain the catastrophic natural history of the disease. Despite all these findings, radiological data are not the same in all the reported patients diagnosed with H-ABC, even if they present the same mutation, so H-ABC gives rise to a spectrum of clinical signs whose pathophysiology still needs to be understood.

## Data Availability Statement

The raw data supporting the conclusions of this article will be made available by the authors, without undue reservation.

## Ethics Statement

The studies involving human participants were reviewed and approved by Institutional committee of bioethics in research of the University of Guanajuato. Written informed consent to participate in this study was provided by the participants' legal guardian/next of kin. The animal study was reviewed and approved by Institutional committee of bioethics in research of the University of Guanajuato and the Benemérita Universidad Autónoma de Puebla.

## Author Contributions

AL-J, AG-V, and VHH contributed to conception and design of the study. AL-J, AG-V, and MA performed the volumetric analysis. JA-J performed the gait experiments. AK-A, AL-J, and VHH organized the database. AL-J and MA performed the statistical analysis. AL-J and VHH wrote the first draft of the manuscript. JE and CC conceived and designed the gait experiments. JA-J performed the gait experiments and JE, CC, and JA-J wrote the ataxic gait section. All authors contributed to manuscript revision and read and approved the submitted version.

## Conflict of Interest

The authors declare that the research was conducted in the absence of any commercial or financial relationships that could be construed as a potential conflict of interest.

## References

[1] van der Knaap MS Naidu S Pouwels PJW Bonavita S van Coster R Lagae L. New syndrome characterized by hypomyelination with atrophy of the basal ganglia and cerebellum. AJNR Am J Neuroradiol. (2002) 23:1466–74. 12372733 PMC7976795

[2] Keays DA Tian G Poirier K Huang G-J Siebold C Cleak J. Mutations in alpha-tubulin cause abnormal neuronal migration in mice and lissencephaly in humans. Cell. (2007) 128:45–57. 10.1016/j.cell.2006.12.017 17218254 PMC1885944

[3] Jaglin XH Poirier K Saillour Y Buhler E Tian G Bahi-Buisson N. Mutations in the beta-tubulin gene TUBB2B result in asymmetrical polymicrogyria. Nat Genet. (2009) 41:746–52. 10.1038/ng.380 19465910 PMC2883584

[4] Chakraborti S Natarajan K Curiel J Janke C Liu J. The emerging role of the tubulin code: from the tubulin molecule to neuronal function and disease. Cytoskeleton (Hoboken). (2016) 73:521–50. 10.1002/cm.21290 26934450

[5] Janke C Magiera MM. The tubulin code and its role in controlling microtubule properties and functions. Nat Rev Mol Cell Biol. (2020) 21:307–26. 10.1038/s41580-020-0214-3 32107477

[6] Search Results. HUGO Gene Nomenclature Committee. Available online at: https://www.genenames.org/tools/search/#!/genes?query=tubulin%20or%20%22tubulin%22&start=0&rows=10 (accessed March 30, 2020).

[7] Leandro-García LJ Leskelä S Landa I Montero-Conde C López-Jiménez E Letón R. Tumoral and tissue-specific expression of the major human beta-tubulin isotypes. Cytoskeleton (Hoboken). (2010) 67:214–23. 10.1002/cm.20436 20191564

[8] Gonçalves FG Freddi TdAL Taranath A Lakshmanan R Goetti R Feltrin FS. Tubulinopathies. Top Magn Reson Imaging. (2018) 27:395–408. 10.1097/RMR.0000000000000188 30516692

[9] Curiel J Rodríguez Bey G Takanohashi A Bugiani M Fu X Wolf NI. TUBB4A mutations result in specific neuronal and oligodendrocytic defects that closely match clinically distinct phenotypes. Hum Mol Genet. (2017) 26:4506–18. 10.1093/hmg/ddx338 28973395 PMC7462055

[10] van der Knaap MS Linnankivi T Paetau A Feigenbaum A Wakusawa K Haginoya K. Hypomyelination with atrophy of the basal ganglia and cerebellum: follow-up and pathology. Neurology. (2007) 69:166–71. 10.1212/01.wnl.0000265592.74483.a6 17620549

[11] Ferreira C Poretti A Cohen J Hamosh A Naidu S. Novel TUBB4A mutations and expansion of the neuroimaging phenotype of hypomyelination with atrophy of the basal ganglia and cerebellum (H-ABC). Am J Med Genet A. (2014) 164A:1802–1807. 10.1002/ajmg.a.36526 24706558 PMC10506160

[12] Joyal KM Michaud J van der Knaap MS Bugiani M Venkateswaran S. Severe TUBB4A-related hypomyelination with atrophy of the basal ganglia and cerebellum: novel neuropathological findings. J Neuropathol Exp Neurol. (2019) 78:3–9. 10.1093/jnen/nly105 30476126

[13] Lopez-Juarez A Gonzalez-Vega A Kleinert-Altamirano A Piazza V Garduno-Robles A Alata-Tejedo MI. Auditory impairment in H-ABC tubulinopathy. J Comp Neurol. (2021) 529:957–68. 10.1002/cne.24990 32681585

[14] Mercimek-Mahmutoglu S van der Knaap MS Baric I Prayer D Stoeckler-Ipsiroglu S. Hypomyelination with atrophy of the basal ganglia and cerebellum (H-ABC). Report of a new case. Neuropediatrics. (2005) 36:223–6. 10.1055/s-2005-865715 15944912

[15] Pizzino A Pierson TM Guo Y Helman G Fortini S Guerrero K. TUBB4A *de novo* mutations cause isolated hypomyelination. Neurology. (2014) 83:898–902. 10.1212/WNL.0000000000000754 25085639 PMC4153852

[16] Purnell SM Bleyl SB Bonkowsky JL. Clinical exome sequencing identifies a novel TUBB4A mutation in a child with static hypomyelinating leukodystrophy. Pediatr Neurol. (2014) 50:608–11. 10.1016/j.pediatrneurol.2014.01.051 24742798 PMC4029864

[17] Bologna M Berardelli A. Cerebellum: an explanation for dystonia? Cerebellum Ataxias. (2017) 4:6. 10.1186/s40673-017-0064-8 28515949 PMC5429509

[18] Garduno-Robles A Alata M Piazza V Cortes C Eguibar JR Pantano S. Features in a rat model of H-ABC tubulinopathy. Front Neurosci. (2020) 14:555. 10.3389/fnins.2020.00555 32581692 PMC7284052

[19] GAREY LJ. Atlas of the human brain. J Anat. (1997) 191:477–8. 10.1046/j.1469-7580.1997.191304773.x

[20] Kalanjati VP. Estimation of cranial capacity and growth indicators in elementary school children. Int. J. Morphol. (2014) 32:07–11. 10.4067/S0717-95022014000100001

[21] Thompson DK Matthews LG Alexander B Lee KJ Kelly CE Adamson CL. Tracking regional brain growth up to age 13 in children born term and very preterm. Nat Commun. (2020) 11:696. 10.1038/s41467-020-14334-9 32019924 PMC7000691

[22] Hamilton EM Polder E Vanderver A Naidu S Schiffmann R Fisher K. Hypomyelination with atrophy of the basal ganglia and cerebellum: further delineation of the phenotype and genotype–phenotype correlation. Brain. (2014) 137:1921–30. 10.1093/brain/awu110 24785942 PMC4345790

[23] Miyatake S Osaka H Shiina M Sasaki M Takanashi J-I Haginoya K. Expanding the phenotypic spectrum of TUBB4A-associated hypomyelinating leukoencephalopathies. Neurology. (2014) 82:2230–7. 10.1212/WNL.0000000000000535 24850488

[24] Erro R Hersheson J Ganos C Mencacci NE Stamelou M Batla A. H-ABC syndrome and DYT4: variable expressivity or pleiotropy of TUBB4 mutations? Mov Disord. (2015) 30:828–33. 10.1002/mds.26129 25545912

[25] Tonduti D Aiello C Renaldo F Dorboz I Saaman S Rodriguez D. TUBB4A-related hypomyelinating leukodystrophy: new insights from a series of 12 patients. Eur J Paediatr Neurol. (2016) 20:323–30. 10.1016/j.ejpn.2015.11.006 26643067

[26] Kancheva D Chamova T Guergueltcheva V Mitev V Azmanov DN Kalaydjieva L. Mosaic dominant TUBB4A mutation in an inbred family with complicated hereditary spastic paraplegia. Mov Disord. (2015) 30:854–8. 10.1002/mds.26196 25772097

[27] Mongerson CRL Jaimes C Zurakowski D Jennings RW Bajic D. Infant corpus callosum size after surgery and critical care for long-gap esophageal atresia: qualitative and quantitative MRI. Sci Rep. (2020) 10:6408. 10.1038/s41598-020-63212-3 32286423 PMC7156662

[28] Holmgren B Urbá-Holmgren R Riboni L Vega-SaenzdeMiera EC. Sprague Dawley rat mutant with tremor, ataxia, tonic immobility episodes, epilepsy and paralysis. Lab Anim Sci. (1989) 39:226–8. 2724922

[29] Duncan ID Lunn KF Holmgren B Urba-Holmgren R Brignolo-Holmes L. The *taiep* rat: a myelin mutant with an associated oligodendrocyte microtubular defect. J Neurocytol. (1992) 21:870–84. 10.1007/BF01191684 1469463

[30] Duncan ID Bugiani M Radcliff AB Moran JJ Lopez-Anido C Duong P. mutation in the Tubb4a gene leads to microtubule accumulation with hypomyelination and demyelination. Ann Neurol. (2017) 81:690–702. 10.1002/ana.24930 28393430 PMC5495199

[31] Brusse E Maat-Kievit JA van Swieten JC. Diagnosis and management of early- and late-onset cerebellar ataxia. Clin Genet. (2007) 71:12–24. 10.1111/j.1399-0004.2006.00722.x 17204042

[32] Fogel BL. Childhood cerebellar ataxia. J Child Neurol. (2012) 27:1138–45. 10.1177/0883073812448231 22764177 PMC3490706

[33] Hadjivassiliou M Martindale J Shanmugarajah P Grünewald RA Sarrigiannis PG Beauchamp N. Causes of progressive cerebellar ataxia: prospective evaluation of 1500 patients. J Neurol Neurosurg Psychiatry. (2017) 88:301–9. 10.1136/jnnp-2016-314863 27965395

[34] Manto M Marmolino D. Cerebellar ataxias. Curr Opin Neurol. (2009) 22:419–29. 10.1097/WCO.0b013e32832b9897 19421057

[35] Bugiani M Vuong C Breur M van der Knaap MS. Vanishing white matter: a leukodystrophy due to astrocytic dysfunction. Brain Pathol. (2018) 28:408–21. 10.1111/bpa.12606 29740943 PMC8028328

[36] Neychev VK Gross RE Lehéricy S Hess EJ Jinnah HA. The functional neuroanatomy of dystonia. Neurobiol Dis. (2011) 42:185–201. 10.1016/j.nbd.2011.01.026 21303695 PMC3478782

[37] Prudente CN Hess EJ Jinnah HA. Dystonia as a network disorder: what is the role of the cerebellum? Neuroscience. (2014) 260:23–35. 10.1016/j.neuroscience.2013.11.062 24333801 PMC3928686

[38] Shakkottai VG Batla A Bhatia K Dauer WT Dresel C Niethammer M. Current opinions and areas of consensus on the role of the cerebellum in dystonia. Cerebellum. (2017) 16:577–94. 10.1007/s12311-016-0825-6 27734238 PMC5336511

[39] Barzilay E Bar-Yosef O Dorembus S Achiron R Katorza E. Fetal brain anomalies associated with ventriculomegaly or asymmetry: an MRI-based study. AJNR Am J Neuroradiol. (2017) 38:371–5. 10.3174/ajnr.A5009 28059712 PMC7963819

[40] Ikuta T Mizobuchi M Katayama Y Yoshimoto S Ioroi T Yamane M. Evaluation index for asymmetric ventricular size on brain magnetic resonance images in very low birth weight infants. Brain Dev. (2018) 40:753–9. 10.1016/j.braindev.2018.05.007 29807844

[41] Oh KY Gibson TJ Pinter JD Pettersson D Shaffer BL Selden NR. Clinical outcomes following prenatal diagnosis of asymmetric ventriculomegaly, interhemispheric cyst, and callosal dysgenesis (AVID). Prenat Diagn. (2019) 39:26–32. 10.1002/pd.5393 30511781

