# Supplementary material for: Longitudinal Evaluation of Cerebellar Signs of H-ABC Tubulinopathy in a Patient and in the taiep Model
Source: Front Neurol. 2021 Jul 14;12:702039. doi: 10.3389/fneur.2021.702039 (PMC8317997; doi:10.3389/fneur.2021.702039)
Supplement: Supplementary file 1 [file Data_Sheet_1.docx]

Supplementary Material

# Supplementary Data

**Video 1**

Video ST1_11YF.mp4
Video of the T1 weighed image in the sagittal section of the entire brain of the patient at the age of 11, consisting of 15 slices. Made with the free and open source code software tool Horos (<https://horosproject.org/>)

**Video 2**
Video ST1_5YF.mp4
Video of the T1 weighed image in the sagittal section of the entire brain of the patient at the age of 5, consisting of 15 slices. Made with the free and open source code software tool Horos (<https://horosproject.org/>)

**Video 3**
Video AF11YF.mp4
Video of the FLAIR image in the axial section of the entire brain of the patient at the age of 11, consisting of 15 slices. Made with the free and open source code software tool Horos (<https://horosproject.org/>)

**Video 4**

Video AF5YF.mp4
Video of the FLAIR image in the axial section of the entire brain of the patient at the age of 5, consisting of 15 slices. Made with the free and open source code software tool Horos (<https://horosproject.org/>)

**Video 5.**

**Representative gait pattern of a WT rat at three months used to calculate the step pattern and the regularity index**. The system records each step to make a spatio-temporal analysis. Below, is a graphic representation of the step sequence pattern. WT animals show an AB pattern (LF, RH, RF, LH). LF: left front; RH: right hind; RF: right front; LH: left hind.

**Video 6.**

**Representative gait pattern of a *taiep* rat used to calculate the step pattern and the regularity index.** The system records each step to make a spatio-temporal analysis.  Below, is a graphic representation of the step sequence pattern. *Taiep* animals show a decrease in the number of complete stepping cycles, combining AB (LF, RH, RF, LH) and RA (rotary) patterns (RF, LF,RH, LH). LF: left front; RH: right hind; RF: right front; LH: left hind.


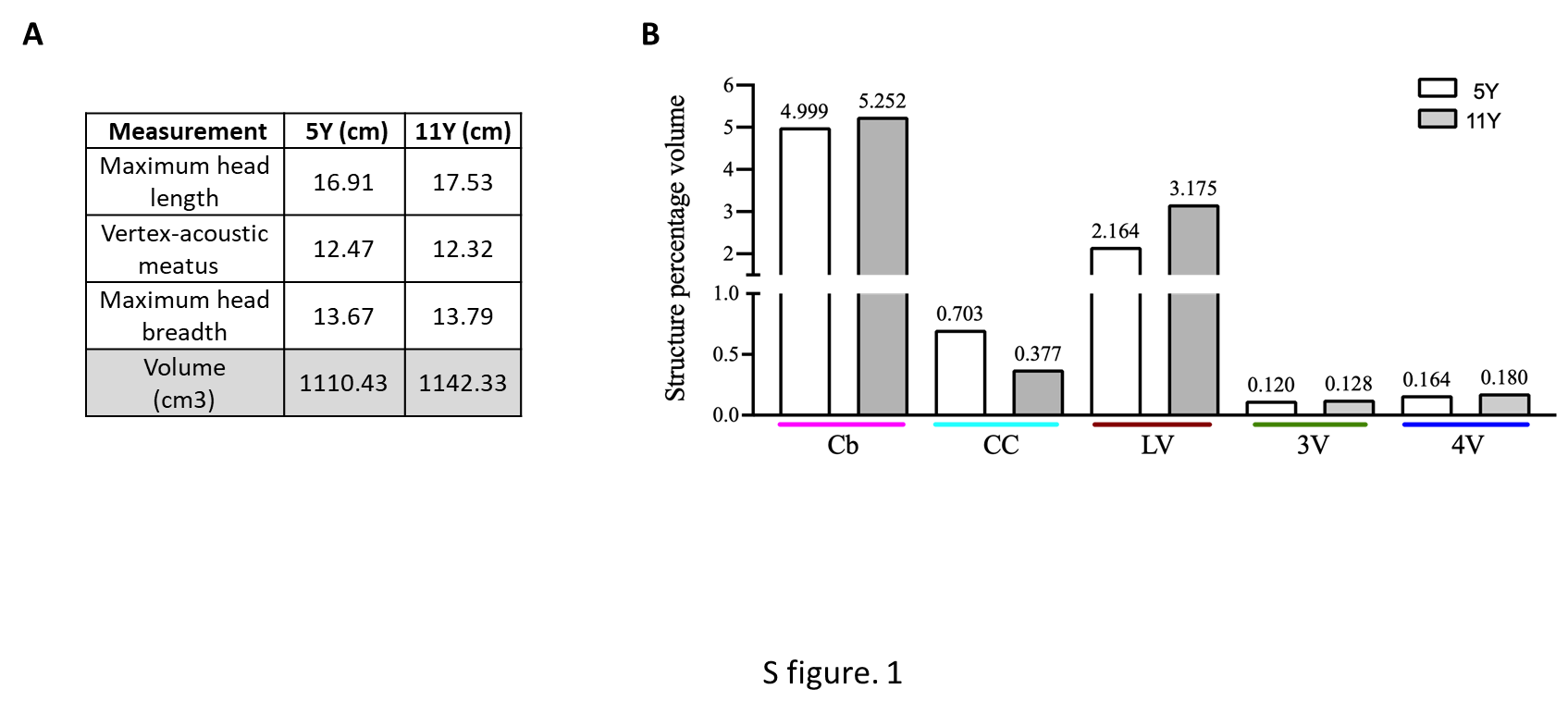


**Supplementary Figure 1. Semiquantitative analysis of cerebellum, corpus callosum and ventricles volume in the patient at ages 5 and 11**. Table shows cranial values used to calculate cranial volume in the H-ABC patient. The volume of each structure of interest is represented as the percentage of its occupancy into the cranial cavity (B).


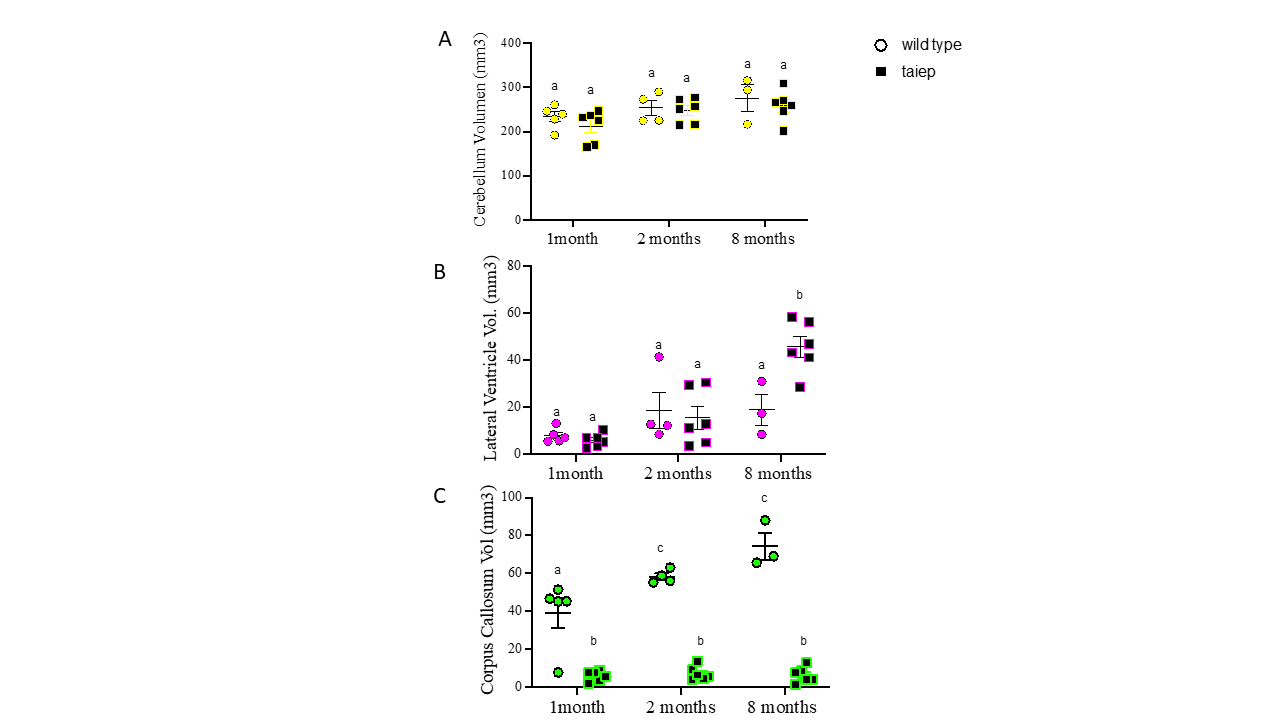


**Supplementary Fig 2. Quantitative analysis of volume (mm3) of cerebellum, corpus callosum and lateral ventricles.**  Scatter plot of the values of encephalic volume of cerebellum, corpus callosum and lateral ventricles. Non-significant differences were obtained for the cerebellum volume of WT and *taiep* rats along the analyzed periods **(A)**. Cc volume of *taiep* rats remains smaller than cc WT volume at all analyzed ages (**B**). Lateral ventricle volume increases significantly compared to LV WT volume at 8 months (**C**). The data obtained for each rat are represented by one point in the plot (at least n=3 for group). Two-way ANOVA followed by Tukey’s multiple comparison test was performed. Different letters indicate significant differences between groups (**A**-**C**).
